# Systemic vascular phenotypes of Loeys-Dietz syndrome in a child carrying a de novo R381P mutation in TGFBR2: a case report

**DOI:** 10.1186/1756-0500-6-456

**Published:** 2013-11-12

**Authors:** Kiyoshi Uike, Yuki Matsushita, Yasunari Sakai, Osamu Togao, Michinobu Nagao, Yoshito Ishizaki, Hazumu Nagata, Kenichiro Yamamura, Hiroyuki Torisu, Toshiro Hara

**Affiliations:** 1 Department of Pediatrics, Kyushu University, Fukuoka 812-8582, Japan; 2 Department of Clinical Radiology, Kyushu University, Fukuoka 812-8582, Japan; 3 Department of Molecular Imaging and Diagnosis, Graduate School of Medical Sciences, Kyushu University, Fukuoka 812-8582, Japan

**Keywords:** Loeys–Dietz syndrome, Transforming growth factor-beta receptor 2 (*TGFBR2*), Vascular phenotypes, Magnetic resonance imaging (MRI)

## Abstract

**Background:**

Loeys–Dietz syndrome, also known as Marfan syndrome type II, is a rare connective tissue disorder caused by dominant mutations in *transforming growth factor*-*beta receptors* (*TGFBR1* and *2*).

**Case presentation:**

We report a 7-year-old Japanese boy with Loeys–Dietz syndrome who carried a novel, *de novo* missense mutation in *TGFBR2* (c.1142g > c, R381P). He showed dysmorphic faces and skeletal malformations that were typical in previous cases with Loeys-Dietz syndrome. The cardiac studies disclosed the presence of markedly dilated aortic root and patent ductus aorteriosus. The cranial magnetic resonance imaging (MRI) and angiography (MRA) detected the tortuous appearances of the bilateral middle cerebral and carotid arteries.

**Conclusion:**

This study depicts the systemic vascular phenotypes of a child with Loeys–Dietz syndrome that were caused by a novel heterozygous mutation of *TGFR2*. A large cohort with serial imaging studies for vascular phenotypes will be useful for delineating the genotype-phenotype correlations of Loeys–Dietz syndrome.

## Background

Loeys–Dietz syndrome is a rare congenital disorder characterized by loose connective tissue [1,2]. As a variant form of Marfan syndrome, affected individuals exhibit progressive vascular diseases such as dilation of the aortic root, aortic dissection and valvular insufficiency [1-3]. In addition to these common phenotypes, patients with Loeys–Dietz syndrome manifest distinct anomalies, such as ocular hypertelorism, high-arched palate, bifid uvula, scoliosis and clubfoot [1,2].

Vascular complications in Loeys–Dietz syndrome are known to progress more rapidly than those in Marfan syndrome [2,3]. Hence the precise diagnosis of Loeys–Dietz syndrome is a critical issue in the long-term management of affected individuals. In this report, we present a Japanese boy with Loeys–Dietz syndrome who carried a novel mutation in the *TGFBR2* gene. This study highlights the moderate to severe vascular phenotypes that resulted from an R381P mutation in *TGFBR2*.

## Methods

MR imaging was performed using a 3.0T system (Intera Achieva; Philips Medical Systems, Best, Netherlands). Polymerase chain reaction (PCR) amplifying the coding exons of *TGFBR2* (chr3:30647994–30735633) was performed as previously described [1,2,4]. Sequencing reactions were performed with the BigDye® Terminator v3.1 cycle sequencing kit (Life Technologies, Grand Island, NY, U.S.A.) according to the manufacturer’s protocol.

## Case Presentation

**Figure 1:**
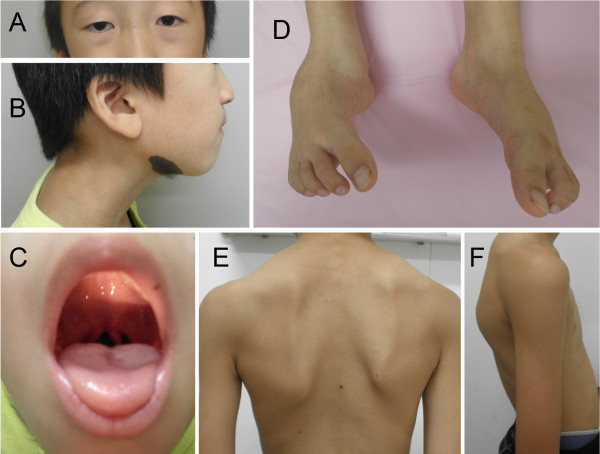
**Dysmorphic features of the present case.** The patient shows the typical facial appearance for Loeys-Dietz syndrome with ocular hypertelorism and mild strabismus **(A)**. Other symptoms include ectopic hair growth on the right mandible **(B)**, high-arched cleft palate with bifid uvula **(C)**, bilateral clubfoot (**D**), and kyphoscoliosis **(E, F)**.

**Figure 2:**
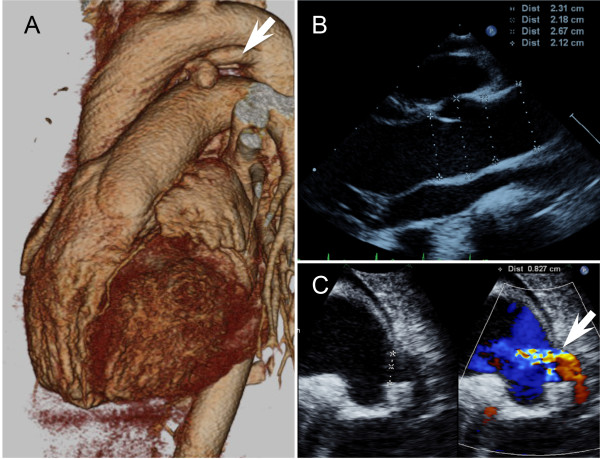
**Cardiovascular phenotypes of the present case.** Imaging studies from a 3D-reconstructed CT scan **(A)** and echocardiographies **(B and C)** are shown. The case had abnormally dilated the aortic root **(A, B)** and the patent ductus arteriosus (arrows in **A** and **C**). Note that, in panel **C**, the shunt flows are present across the ductus.

**Figure 3:**
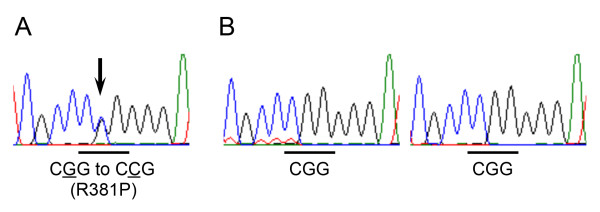
**The *de novo *mutation at *TGFBR2 *exon 5.** The sequence chromatograms show the heterozygous mutation in *TGFBR2* (c.1142g > c) in the proband’s DNA **(A)**. The arrow indicates the position of the missense mutation replacing Arg381 (cgg) with Pro (ccg). The parents of the case **(B)** show the normal *TGFBR2* genotype (left, father; right, mother).

A 7-year-old Japanese boy born to healthy Japanese parents was referred to our department for assessment of systemic anomalies. He had undergone surgical repairs for right undescended testis, inguinal hernia and right clubfoot. Genetic disorders were suspected from his dysmorphic facial appearance consisting of hypertelorism, ectopic hair formation on the right lower mandible, and bifid uvula (Figure 1A-C). Physical examination also revealed hypermobility of joints (not shown), talipes equinovarus and scoliosis (Figure 1D-F). His intelligence was normal. He had no history of stroke, heart attack or arrhythmia. Ocular examination excluded ectopia lentis. Ehlers-Danlos syndrome or related connective disorders were suspected from these clinical features. Computed tomography (CT) scan revealed protrusion of the aortic arch (Figure 2A) and the presence of a patent ductus arteriosus (Figure 2B). Echocardiography revealed aortic root dilatation to 26.7 mm in diameter (Figure 2C). The dysmorphic features and cardio-vasuclar phenotypes of the present case allowed us to diagnose him as having Loeys–Dietz syndrome. To confirm the genetic diagnosis of Loeys–Dietz syndrome, we sequenced the coding exons of the *TGFBR2* gene. A novel heterozygous missense mutation (c.1142g > c, R381P) was detected in *TGFBR2* exon 5 of the proband (Figure 3A) but not his parents (Figure 3B).

**Figure 4:**
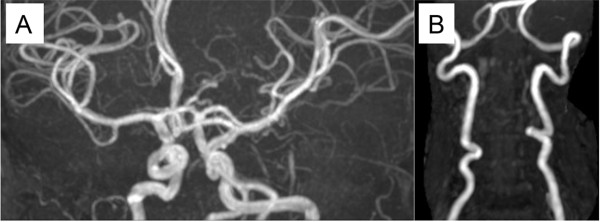
**Neurovascular phenotypes in magnetic resonance imaging.** The cranial and cervical magnetic resonance angiography displays the tortuous appearances of the carotid, middle cerebral **(A)** and vertebral arteries **(B)**.

**Figure 5:**
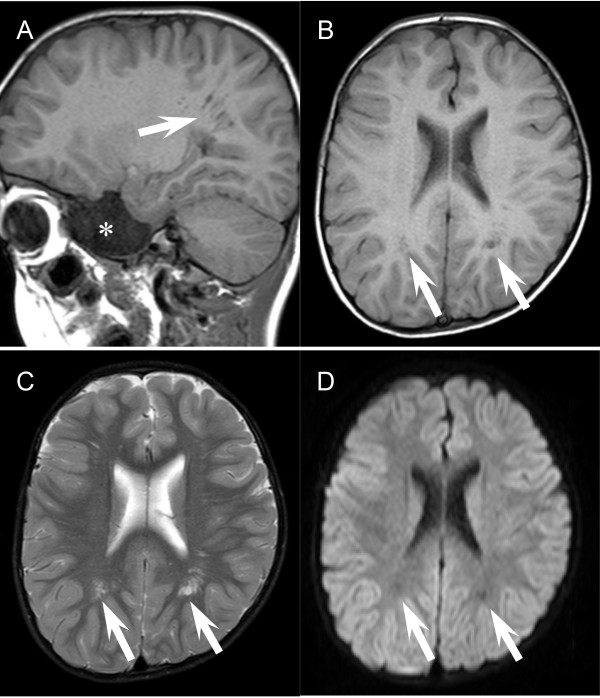
**Enlarged Virchow–Robin spaces in the present case.** The sagittal **(A)** and axial **(B-D)** views of T1- **(A and B)**, T2- **(C)** and diffusion-weighted images **(D)** show the dilated perivascular “Virchow–Robin” spaces (arrows). Note that the dilated perivascular regions exhibit iso-intensity to the fluidal spaces in the lateral ventricle **(A-D)**. Diffusion-weighted images were acquired with the b-factor of 1,000 s/mm^2^. Asterisk **(A)** denotes the presence of subarachnoid cyst at the right middle cranial fossa.

To assess systemic vascular malformation, we performed cranial and cervical magnetic resonance imaging (MRI) studies. The MR angiographies revealed the prominently tortuous appearance of the vertebral, carotid and middle cerebral arteries (Figure 4A and B). These findings were consistent with those in previous reports [5,6]. No vascular aneurysm was identified in the cervical and cranial MRI. Of note, we found in the cranial MRI that unusually enlarged perivascular spaces at the bilateral posterior white matter without ischemic damage, hemorrhagic lesions or metabolic degeneration (Figure 5A-C). Spinal MRI detected enlarged sacral foramina as typically observed in Marfan syndrome and Loeys–Dietz syndrome (data not shown).

## Conclusions

This report presents the systemic phenotypes of Loeys–Dietz syndrome that were caused by a novel *TGFBR2* mutation (R381P) in a Japanese case. The systemic vascular phenotypes of this case indicate the strong penetrance as well as the dominant effects of the heterozygous missense mutation.

The *TGFBR2* gene encodes transforming growth factor-beta receptor II (70/80 kDa), a membrane-bound, serine/threonine kinase domain-containing protein. This protein forms a heterodimeric complex with another receptor protein, thereby activating the downstream signaling molecules, such as SMAD proteins, upon stimulation by TGF-beta ligands. Previous studies showed that the majority of pathogenic *TGFBR2* mutations were identified in the exons encoding the protein kinase domain [1-3]. *In vitro* studies suggested that such mutations disturbed not only the kinase activity of TGFBR2, but also internalization process of the receptor, suggesting the dominant effects of heterozygous mutations. Given that the R381P mutation in this case (Figure 3A) was also located at the kinase domain of TGFBR2, we speculated that the mutation caused deleterious effects on both its kinase activity and the receptor internalization. An angiotensin II receptor antagonist, losartan, has been recently shown to down-regulate the expression of TGFBRs, and thus proved to be effective in preventing the progressive aortic dilation and development of aneurysm in Marfan and Loeys-Dietz syndromes. A large cohort, including the present case, is thus needed to monitor its efficacy and side effects, thereby elucidating prognostic factors and contraindications of the therapies.

It is well known that prevention of aortic dilation is a vital issue for the long-term survival of patients with Loeys-Dietz syndrome. On the other hand, there are no reports clearly demonstrating the association of Marfan or Loeys-Dietz syndrome with neurovascular complications [7,8]. Nonetheless, recent reports showed sporadic cases with intracranial aneurysm as well as tortuosity of carotid arteries in this disorder [7,9,10]. Given the progressive nature of the cardiovascular phenotypes of Loeys–Dietz syndrome, one could argue that only severely affected cases may experience such late-onset neurovascular complications. It also remains to be determined whether the enlarged perivascular lumens in this case (Figure 5A-C) are associated with the neurovascular phenotypes of Loeys-Dietz syndrome. Future studies will address the issues of their genotype-phenotype correlations, particularly on their comorbidity with neurovascular complications. Large cohorts with serial imaging studies can be considered to determine whether certain genotypes may correlate to their specific phenotypes.

## Consent

This study was conducted in compliance with the institutional review board at Kyushu University Hospital. Written informed consent was obtained from the patient’s parents for publication of this Case Report and any accompanying images. A copy of the written consent is available for review by the Editor-in-Chief of this journal.

## Competing interests

The authors declare that they have no competing interests.

## Authors’ contributions

KU and YM wrote the first draft of the manuscript. KU, YM, and YS conducted the experiments. YS and TH directed the study, supervised experiments and edited the manuscript. OT and MN supervised and confirmed the radiological findings. KU, HN, and KY assessed the cardiovascular complications. KU, KU, YS, YI, HN, KY, and HT managed the patient. All authors read and approved the final manuscript.
